# Incidental Normocalcemic Primary Hyperparathyroidism Presenting With Symptomatic Hypophosphatemia: A Case Report

**DOI:** 10.7759/cureus.44378

**Published:** 2023-08-30

**Authors:** Omar Tabbikha, Joanne Chamy, Michael El Khoury

**Affiliations:** 1 General Surgery, Faculty of Medicine and Medical Sciences at University of Balamand, Beirut, LBN; 2 Vascular and Endovascular Surgery, Haykel Hospital/Lebanese University, Beirut, LBN; 3 General Surgery, Haykel Hospital/University of Balamand, Beirut, LBN

**Keywords:** secondary hyperparathyroidism, symptomatic hypophosphatemia, parathyroidectomy, parathyroid adenoma, normocalcemic hyperparathyroidism

## Abstract

Normocalcemic primary hyperparathyroidism (NHPT) is a newly defined variant of primary hyperparathyroidism (PHPT). It’s defined by consistently normal total and ionised calcium levels with elevated parathyroid hormone in the absence of secondary causes of hyperparathyroidism in at least three consecutive times over a period of three to six months. Consensus whether the same criteria used to recommend surgery in PHPT should be used to recommend surgery in NHPT is still lacking. Even though PHPT is known to cause hypophosphatemia, serum phosphate is not relevant when diagnosing it or NHPT. No current guideline include any phosphate cutoff level to guide management or indicate surgery in PHPT or NHPT patients. Herein, we present a rare case of incidental NHPT presenting with symptomatic hypophosphatemia and managed surgically.

## Introduction

Primary hyperparathyroidism (PHPT) is defined biochemically by hypercalcemia with elevated or inappropriately normal parathyroid hormone (PTH) levels as a result of parathyroid gland overactivity [1]. Although the clinical presentation of PHPT is highly variable, asymptomatic hypercalcemia detected incidentally is the most common presentation [2]. Normocalcemic primary hyperparathyroidism (NHPT) is a newly defined variant of PHPT which has led to new questions regarding its diagnosis and management [3]. In contrast to PHPT, NPHP is characterised by consistently normal total and ionised calcium (Ca) levels with elevated PTH in the absence of secondary causes of hyperparathyroidism [4]. PHPT is also known to cause mild hypophosphatemia especially in severe cases [5]; however, no current guidelines include any phosphate (P) cutoff level to diagnose or indicate surgery in PHPT [1] or NPHT patients. To our knowledge, the clinical correlation between P and PHPT has been investigated to a small extent in the literature, while that between P and NHPT has not been described at all. Herein, we present a rare case of a patient with incidental NHPT presenting with symptomatic hypophosphatemia.

This article was presented as a poster at the University of Balamand First Medical Congress 2023: Updates in Medicine on April 29, 2023.

## Case presentation

A 44-year-old female patient with a history of nontoxic multinodular goitre presented to our institution for a follow-up on her thyroid nodules. The patient had undergone fine needle aspiration of thyroid nodules eight months prior to presentation and they were benign in nature. The patient had normal vitals in the current presentation, was still asymptomatic, and had no subjective or objective changes in goitre size. She had previously undergone only one surgery, which was a hysterectomy for uterine fibroids. She had no allergies, was not a smoker nor an alcohol consumer, and had no pertinent family medical history. To note, the patient was not taking any medications.

**Figure 1:**
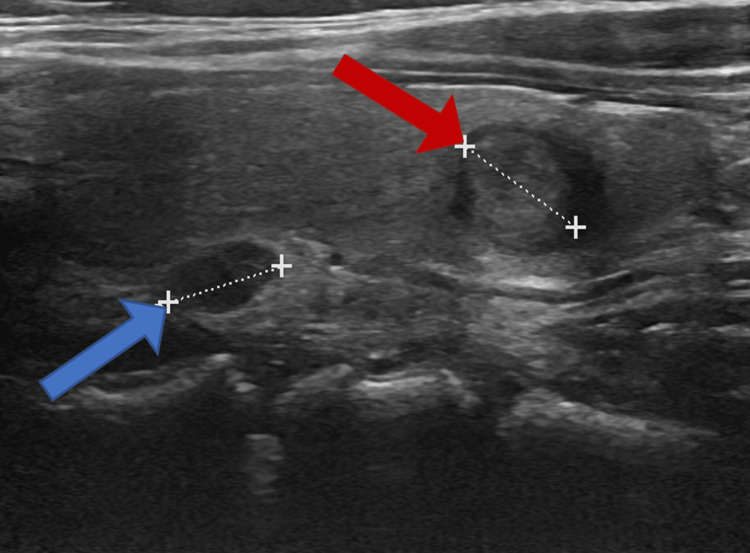
Left thyroid ultrasound showing a 0.8cm posterior thyroid nodule suggestive of parathyroid adenoma (blue arrow) and one of the many unchanged thyroid nodules, 0.9cm in size (red arrow)

Thyroid USG was done for reevaluation and grossly unchanged bilateral thyroid nodules were noted with the most suspicious being an unchanged 1 cm nodule in the left hemithyroid. Nevertheless, a 0.8 cm well-circumscribed nodule on the posterior surface of the left hemithyroid, suggestive of the parathyroid nodule was noted this time on the USG (Figure 1).

**Figure 2:**
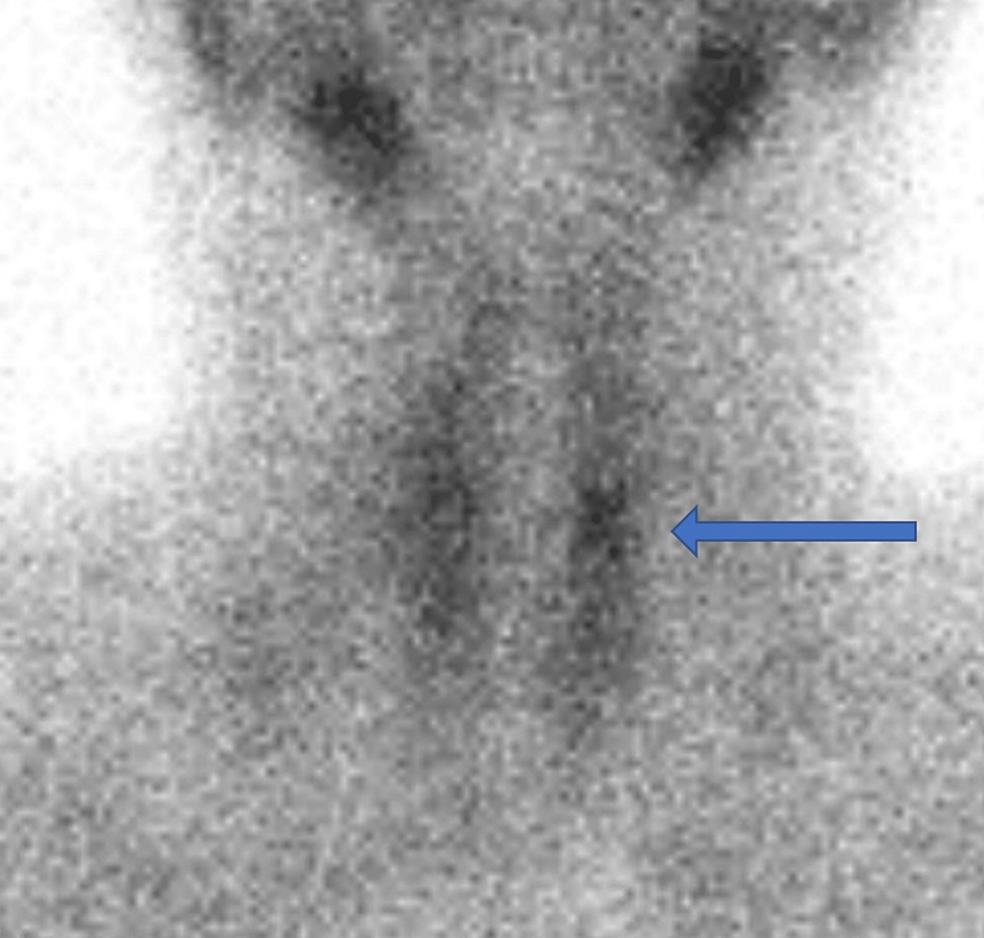
Neck anterior image showing persistent focal increase of radio-tracer uptake in the mid aspect of the left hemithyroid (blue arrow) two hours after the intravenous administration of 19.8 mCi of 99mTc-Sestamibi, which is consistent with left parathyroid adenoma

Labs were done for further evaluation and the patient was found to have normal serum Ca and P levels of 9.4 mg/ dL and 4.1 mg/ dL, respectively, with mildly elevated PTH level 49 pg/ml (normal range: 9.2-44.5 pg/ml) and low vitamin D level of 16.5 ng/ml with no other pertinent laboratory derangement (Table 1). A sestamibi scan was ordered and its findings were compatible with left parathyroid adenoma (Figure 2).

**Table 1:** Patient’s pertinent laboratory values at different stages of her presentation Ca: calcium; P: phosphate; Vit-D: vitamin D; PTH: parathyroid hormone

Initial presentation				Emergency Room presentation			Prior to surgery			Intraoperative		
Ca: 9.4 mg/dl	P: 4.1 mg/dl	PTH: 49 pg/ml	Vit-D: 16.5 to 35.4 ng/ml	Ca: 8.7 mg/dl	P: 1.3 mg/dl	PTH: 108.2 pg/ml	Ca: 9 mg/dl	P: 3.7 mg/dl	PTH: 64 pg/ml	PTH incision: 46 pg/ml	PTH 10 minutes: 42 pg/ml	PTH 20 minutes: 25 pg/ml

The patient was asked about symptoms that could suggest a history of PHPT or secondary hyperparathyroidism (SHPT) other than the vitamin D deficiency that could explain this normocalcemic hyperparathyroidism. She denied any symptom related to hypercalcemia as in bone pain, polyuria, nephrolithiasis, or constipation, nor any symptom related to hypocalcemia as in tingling or muscle spasms; moreover, she denied any history of previous osteoporosis or fractures and dual x-ray absorptiometry (DEXA) scan was done and showed normal lumbar and forearm result with mild osteopenia in the hip. The patient has no previous symptoms suggestive of hypophosphatemia as in myalgia and muscle weakness. She has no symptoms suggestive of dietary deficiency and no family history of any electrolyte abnormality that could suggest inborn errors of metabolism. 

The patient was started on vitamin D supplementation and her value increased to 35.4 ng/ml in two months. She was planned to have continuous follow-ups on her thyroid nodules and to repeat labs in three to six months. However, three months later the patient presented to our emergency department with severe fatigue, muscle weakness, bone pain, myalgia, and one episode of vomiting of same day duration. She denied fever, cough, dyspnea, chest pain, palpitation, or diarrhoea. Her vital signs were normal and on physical exam, she was awake, alert, and oriented to time, place, and person. She had a normal cardiac rhythm with good bilateral air entry. She had a soft, non-tender, non-distended abdomen with a negative Murphy sign. Laboratory tests were done and they showed normal complete blood count (CBC) and creatinine with estimated glomerular filtration rate (eGFR) of 96.62 ml/min/1.73m^2^. She had normal electrolytes except for mild hypokalemia (3.3 mg/dL) and moderate hypophosphatemia (1.3 mg/dL). The patient was also found to have normal total serum Ca (8.7 mg/dL) with elevated PTH (108.2 pg/ml) (Table 1).

The patient was diagnosed with moderate symptomatic hypophosphatemia in the context of possible NHPT and was admitted for monitoring and electrolyte replacement. The patient was hydrated and took runs of magnesium, potassium, and phosphate and her potassium level increased to 4 mg/dL and P level to 3.06 mg/dL the next day, post transfusions. The possible treatment options of continuing conservative management with further investigations versus surgical management were discussed with the patient and her endocrinologist. She decided to undergo total thyroidectomy with parathyroid adenoma resection at the same time since she didn’t want to keep on following up on her thyroid nodules and was disturbed by her goitre appearance; moreover, she didn’t want to keep on monitoring her parathyroid adenoma and was worried about developing another episode of hypophosphatemia since it was attributed to it.

Surgery was done under general anaesthesia and with a collar incision. We started with bilateral neck exploration and only one enlarged parathyroid gland (left inferior) was identified, excised, and sent as frozen pathology, which showed hypercellular parathyroid suggestive of parathyroid adenoma. The surgery was continued with total thyroidectomy, which was not complicated and the other three parathyroid glands were preserved. To note, the PTH value two days prior to surgery during preoperative evaluation was found to be 64 pg/ml, Ca was also normal at 9 mg/dL, and P 3.7 mg/dL (Table 1); however, on incision, the value of PTH withdrawn from left upper limb was found to be 46 pg/ml and, 10 minutes after parathyroid gland excision, decreased only to 42 pg/ml and then 20 minutes after excision it reached 25 pg/ml (Table 1). On day one post operation, her Ca level was 8.2 mg/dL and P was 3.7 mg/dL. Her hospital stay was uneventful and she showed no signs of hypocalcemia or dysphonia and was discharged on day one post operation. The final histopathology showed parathyroid adenoma with thyroid showing nodular hyperplasia and no signs of malignancy.

## Discussion

NHPT was first recognised in the Third International Workshop on Parathyroid Disorders in 2008 [1]. Its diagnosis was further defined during the fourth workshop in 2014 [6] as persistently normal serum ionised and total Ca levels with persistently high serum PTH levels, measured at least three consecutive times over a period of three to six months, after secondary causes of increased serum PTH have been excluded [4]. The prevalence of NHPT is highly variable in literature but has been estimated to range between 0.1% and 6% [7]. Even though multiple distinct hypotheses have been proposed, the pathophysiology of NHPT is still unknown [7]. Moreover, whether NHPT will progress toward hypercalcemic PHPT over time is not clear; for instance, a study by Schini and colleagues showed that the evolution rate of NHPT to PHPT ranges from zero to 19% in the literature and therefore it was difficult for them to have a definitive conclusion [8]. Hypophosphatemia, which is known to be present in PHPT is not included in the definition of NHPT the same as it is not included in the definition of PHPT. In our case, the total Ca level was used since the ionised Ca level was not available at our institution. The Ca level was normal at three different times within a period of six months and associated with a constant increase in PTH level and one episode of symptomatic hypophosphatemia.

To complete the diagnosis of NHPT, one should exclude the secondary causes of hyperparathyroidism by thorough clinical and laboratory investigations [4]. The secondary causes include vitamin D deficiency from poor dietary intake, low sunlight exposure, and previous bariatric surgeries producing malabsorption conditions [9]. In our case, the patient maintained her NHPT even after the correction of her vitamin D deficiency; moreover, to discriminate NHPT from vitamin D-deficient normocalcemic SHPT, PFindex was proposed. It’s defined by serum Ca × PTH/P, with Ca and P reported in mmol/L, and PTH in pg/mL. It was found that when PFindex is above 34, NHPT or PHPT is diagnosed instead of vitamin D-deficient SHPT with a sensitivity of 96.9% and a specificity of 97.6% [10]. In our case, the PFindex was found to be 112.32 at her first encounter stressing that her hyperparathyroidism was not caused by her vitamin D deficiency solely but NHPT should be suspected. Hypercalciuria is also considered a causative agent for normocalcemic SHPT; it can result from excess sodium intake, excess tea and coffee consumption, and loop diuretics like furosemide [9]. The last is also not the case for our patient; moreover, she has never complained of any urinary symptoms nor has a history of nephrolithiasis. Eventually, our patient decided to undergo a surgical intervention instead of investigating more with a 24-hour urine Ca test. A special mechanism caused by lithium, not used by our patient, induces PTH release thus normocalcemic SHPT through desensitizing Ca-sensing receptors to Ca. Other causative medications worth of mentioning are bisphosphonates and denosumab - used in osteoporosis treatment [11], all of which are not used by our patient so also won’t explain her NHPT with symptomatic hypophosphatemia.

Being the second most abundant mineral in the human body, phosphate represents about 1% of total body weight. Almost all-natural foods are rich in phosphate as milk, fish, poultry and grains. It is mainly found within the intracellular space whereas less than 1% of its unbound inorganic form is metabolically active within the extracellular space and maintained between 2.5-4.5 mg/dL. This narrow concentration window is due to a complex interplay between intestinal absorption, exchange with intracellular and bone storage pools, and renal tubular reabsorption, relying on multiple factors such as the PTH, vitamin D, fibroblast growth factor 23, and Klotho enzyme. Other than being a structural component of bones, teeth, DNA and RNA, phosphate is an essential mineral for producing energy (ATP), buffering blood, regulating gene transcription and enabling signal transduction of many regulatory pathways [12,13].

 Hypophosphatemia is relatively common and is defined as the following: mild (2-2.5 mg/ dL), moderate (1-2 mg/dL) or severe (less than 1 mg/dL). Its clinical symptoms range from asymptomatic to weakness, bone and muscle pain, rhabdomyolysis and altered mental status. Medically significant hypophosphatemia can occur in refeeding syndrome, severe respiratory alkalosis, severe burns and alcohol withdrawal. Some chronic causes include malnutrition, hyperparathyroidism, Cushing’s syndrome, theophylline intoxication, and vitamin D deficiency [14,15]. Our patient was first asymptomatic having a normal P level of 4.1 mg/dL with a low serum vitamin D level of 16.5 ng/dL when first discovering her parathyroid adenoma, along with normal serum Ca of 9.4 mg/dL. Follow-up was planned along with vitamin D supplementation; three months later, the patient presented with symptomatic normocalcemic hyperparathyroidism (Ca 8.7 mg/dL, PTH 108.2 pg/ml) with moderate hypophosphatemia of 1.3 mg/dL translated clinically into fatigue, severe myalgia and weakness, and improved vitamin D level of 34.5 ng/ml. Thus, in the absence of other causes and after vitamin D level normalization, this sole finding of symptomatic hypophosphatemia was attributed to possible NHPT that was confirmed later on with a third normocalcemic value (Table 1).

Regardless if PHPT is normocalcemic or hypercalcemic, parathyroid surgery remains its only definitive treatment. Indeed, parathyroidectomy is recommended for all patients with symptomatic PHPT or asymptomatic PHPT with age less than 50, creatinine clearance less than 60 ml/min, urinary calcium more than 400 mg/ 24 hours, nephrolithiasis, and osteoporosis. Nevertheless, these guidelines do not include any cutoff for phosphate level and do not recommend surgery for hypophosphatemia regardless if it’s symptomatic or not [16]. Consensus on whether these same criteria, including hypophosphatemia, should be used to recommend surgery in NHPT is still lacking. This is because the diagnosis of NHPT requires strict criteria which the retrospective observational studies done before did not abide by and were very heterogeneous in terms of patient selection [3]. Indeed, these articles, as in the studies investigating post-parathyroidectomy bone density changes [17] and prevention of new or reduction in previous kidney stones [18], did not indicate that parathyroidectomy will improve some of these classical or even non-classical complications of NHPT such as hypertension and cardiovascular morbidity, or even improve quality of life [3]. Therefore, prospective studies must investigate whether surgery has a role in improving NHPT complications and whether hypophosphatemia should be included in the guidelines. Until then, parathyroidectomy for NHPT should be recommended only in a case-by-case approach. In our case, it was our patient’s will to go directly for surgery since she did not want to keep on following up on her thyroid nodules and parathyroid adenoma. 

Few data are available in the literature on the type of parathyroidectomy needed in NHPT such as minimal invasive or bilateral neck exploration (BNE), which is challenging for various reasons. It has already been reported that NHPT is characterised by a higher prevalence of multi-gland disease, smaller gland size, and consequently lower cure rates [19,20]. Therefore, one study concluded that in NHPT routine bilateral neck exploration (BNE) is necessary in addition to intraoperative PTH monitoring (ioPTH) [19], while another study suggested proceeding with BNE only when ioPTH fails to drop more than 50% within 10 minutes from resection of the suspected gland; therefore, to have a lower threshold to convert to BNE since in their series of 119 NHPT patients, the percentage of conversion was 4% versus 13% in PHPT [20]. Nevertheless, BNE comes with various risks and is relatively considered aggressive in the setting of asymptomatic NHPT. Therefore, long-term studies are required not only to clarify the role but also to define the type of surgery in NHPT. In our case, BNE was achieved regardless since the patient was undergoing simultaneous total thyroidectomy and only one enlarged parathyroid gland (left inferior) was identified.

## Conclusions

There is no consensus on the management of NHPT and on whether surgery has a role in combating its symptoms. We presented a rare case of symptomatic hypophosphatemia in a patient diagnosed incidentally with NHPT; however, hypophosphatemia in NHPT whether symptomatic or not, is still not described in the literature and the role of P in both NHPT and PHPT has not been defined that can help in the diagnosis or management. Therefore, further controlled studies are needed to further define the management of NHPT and to elucidate whether hypophosphatemia has a role in its diagnosis and management or not.
